# A Sketch of the History, Nature, Advantages and Processes of the Modern Improved Turkish Bath

**Published:** 1877-04-20

**Authors:** Jno. Stainback Wilson

**Affiliations:** Atlanta


### SOUTHERN MEDICAL RECORD.

VOL. VII. ATLANTA, GA., APRIL 20, 1877. No. 4.

THOMAS S. POWELL, M.D.,

W. T. GOLDSMTIH, M. D.,

R. C. WORD, M. D.,

} EDITORS.

R. C. WORD, Business Manager.

SUBSCRIPTION: TWO DOLLARS PER ANNUM, IN ADVANCE.

☞ All communications, and letters on business connected with THE RECORD for 1877 must be addressed to the Business Manager.

### Original and Selected Articles.

### A SKETCH OF THE HISTORY, NATURE, ADVANTAGES AND PROCESSES OF THE MODERN IMPROVED TURKISH BATH.

By JNO. STAINBACK WILSON, M. D., of Atlanta.

What is now called the Turkish bath existed in some form among the Greeks, Romans, and other nations, as far back as history extends. Used by the ancients as a luxury; but their remarkable health and longevity was greatly due to this. The Turks still use it thus; but not the *hot-air* bath, as we do.

The scientific remedial use of the Turkish bath may be dated only about twenty years ago, when it was introduced into Great Britain by Mr. David Urquhart.

Since then, its use has rapidly extended in Europe and in this country, and has received the endorsement of the most distinguished authorities, profes–sional and non-professional.

This is true of the improved Turkish bath, which differs from that of the Turks in the high and graded temperature of our baths; in the exclusion of the violent and unscientific shampooing as practiced by them; and, above all, in the *dryness* of the air of our improved baths. The Turkish bath, then, is not a steam or vapor bath, but a *dry, hot-air* bath.

If steam is used at all, it is only for the purpose of heating coils of pipe, the heat being radiated from these coils, into the rooms. Whenever vapor is diffused through the rooms, the essential distinguishing feature of the Turkish bath is gone, and such a bath should not be called a Turkish bath, but a Russian, vapor, or steam bath, which is far inferior.

In the Turkish bath the dry air comes in contact not only with the 2,000 square inches of the skin, but with the 20,000 square inches of the lungs; thus giving it great advantages over the steam bath, which retards perspiration, and the air of which is breathed with great difficulty, never reaching the ultimate air-cells which close against the water with which the air is saturated.

Another advantage of the Turkish bath is having a separate room of high temperature for the shampooing.

Another still, is having rooms of *different temperatures*, so that bathers may be brought gradually to higher grades.

To sum up, then, the Turkish bath is superior to any other: 1st. Because the air comes in contact with the whole extensive cell-structure of the lungs. 2d. It can be breathed with ease and positive delight at a temperature of from 150 deg. to 200 deg., while vapor cannot be breathed with ease at any temperature, and does not permeate the lungs. 3d. Vapor retards, instead of favoring, perspiration. 4th. It is impossible to endure in a vapor bath that high heat on which the remedial virtues of the Turkish bath mainly depend.

PROCESSES OF THE TURKISH BATH.

Every Turkish bath should have not less than four large, well-ventilated rooms. These are, the Frigidarium, or cooling room; the Tepidarium—first warm, or preparatory room; the Calidarium—hot, or shampooing-room, and the Lavatorium, or wash-room.

The bather first enters the frigidarium, where he undresses and passes into the tepidarium, the temperature of which ranges from 120 deg. to 140 deg. The object of this room is to solicit a general perspiration without any undue excitement or oppression before going into the next, or hot room. These ends are promoted by the use of warm foot-baths and cold cloths to the head.

When a general moisture appears on the skin the bather is passed into the calidarium, which is heated from 140 deg. to 180 deg. or 200 deg. In this room the sweating and shampooing are done, as the latter should always be performed at a high temperature. For this operation the *hands* are preferable, and nothing else should be used except in exceptional cases. After sweating, shampooing, percussing and soaping the bather in the calidarium, he next passes into the lavatorium. In this room he begins with a warm shower-bath, which is gradually changed to cool, and then cold. This not only cleanses him, but it closes the pores and causes a vigorous reaction. This last important result is always accomplished readily after passing through the hot-air bath. We object to the plunge, because unnecessary, and might, in some cases, be dangerous. The bather next returns to the frigidarium, where he dresses slowly; or, if there is any secondary perspiration, he reclines on a lounge until he is "all right." He then goes out, wonderfully refreshed in mind and body, feeling like a new man.

